# Considerable variation of trough β-lactam concentrations in older adults hospitalized with infection—a prospective observational study

**DOI:** 10.1007/s10096-018-3194-x

**Published:** 2018-01-29

**Authors:** Malini Hatti, Nikolitsa Solomonidi, Inga Odenholt, Johan Tham, Fredrik Resman

**Affiliations:** Clinical Infection Medicine, Department of Translational Medicine, Lund University, Rut Lundskogs gata 3, plan 6, SE20502 Malmö, Sweden

## Abstract

In older adults, few studies confirm that adequate concentrations of antibiotics are achieved using current dosage regimens of intravenous β-lactam antibiotics. Our objective was to investigate trough concentrations of cefotaxime, meropenem, and piperacillin in older adults hospitalized with infection. We included 102 patients above 70 years of age. Total trough antibiotic concentrations were measured and related to suggested target intervals. Information on antibiotic dose, patient characteristics, and 28-day outcomes were collected from medical records and regression models were fitted. Trough concentrations for all three antibiotics exhibited considerable variation. Mean total trough concentrations for cefotaxime, meropenem, and piperacillin were 6.5 mg/L (range 0–44), 3.4 mg/L (range 0–11), and 30.2 mg/L (range 1.2–131), respectively. When a target range of non-species-related breakpoint − 5× non-species-related breakpoint was applied, only 36% of patients had both values within the target range. Regression models revealed that severe sepsis was associated with varying concentration levels and increasing age and diminishing kidney function with high concentration levels. The study was not powered to demonstrate consequences in clinical outcomes. Conclusively, in older adults treated with cefotaxime, meropenem, or piperacillin-tazobactam, trough antibiotic concentrations varied considerably. Better predictors to guide dosing regimens of β-lactam antibiotics or increased use of therapeutic drug monitoring are potential ways to address such variations.

**Electronic supplementary material:**

The online version of this article (10.1007/s10096-018-3194-x) contains supplementary material, which is available to authorized users.

## Introduction

The importance of early, correct antibiotic treatment in bacterial infections is undisputed [1]. Optimizing antibiotic treatment means choosing the correct antibiotic and administering a correct dose to ensure efficacy and to minimize the risk of adverse events. Dosing guidelines for adults are often based on results from middle-aged healthy volunteers, but are generalized to all adults [2].

The most commonly used group of antibiotics for severe infections is beta-lactams. The bactericidal effect of beta-lactams depends on the time of unbound antibiotic concentration above the minimum inhibitory concentration (MIC) for the bacteria, *f*T > MIC [3]. Animal studies suggest that 50–70% *f*T > MIC may be sufficient for penicillins and cephalosporins in treating most infections, and for meropenem, 40% may be sufficient [4]. However, immune-deficient and critically ill patients likely require a longer *f*T > MIC [5–7]. Studies suggest that a *f*T > MIC of 100% is required in mice with neutropenia [8]. In human medicine, there is no consensus on ideal *f*T > MIC targets. Most clinical studies have investigated patients with severe sepsis and have defined targets higher than those suggested from animal models, generally *f*T > MIC of 100% [9].

For most patients, the volume of distribution of beta-lactam antibiotics is considered similar, with the exception of small children and pregnant women [10, 11]. The predominant route of elimination is via the kidneys, and dosing of most beta-lactams is adjusted according to estimated glomerulus filtration rates (eGFR). In certain clinical situations, volatile pharmacokinetics for beta-lactams is well documented, including severe sepsis. In severe sepsis, the volume of distribution increases, mainly due to capillary leakage and fluid resuscitation, and augmented as well as reduced renal clearance is common [2, 12]. Increasing age is not only associated with an increased risk of infection, but age-related changes in organ function also result in difficulties in approximating renal function, making the pharmacokinetics of beta-lactam antibiotics unpredictable [7]. Also, adverse effects related to antibiotics, such as diarrhea, nephrotoxicity, and neurotoxicity, are more common among older adults [7, 13, 14].

Even though a large proportion of antibiotics in hospitals are administered to older adults, information on antibiotic concentration attainment in this group is limited. To fully assess the pharmacokinetic of a beta-lactam in an individual patient, repeated concentration measurements are needed. This is, however, seldom feasible in standard care. A more feasible way to assess *f*T > MIC is to measure total trough concentrations during presumed steady state. In the present investigation, trough concentrations were determined for three beta-lactam antibiotics commonly used for severe infections in the region; cefotaxime, meropenem, and piperacillin-tazobactam, in individuals aged 70 years or more, and hospitalized due to an infection. Associations between concentration levels and clinical predictors as well as outcomes were assessed.

## Materials and methods

### Study setting

This prospective observational study was conducted at Skåne University Hospital Malmö, Skåne county, Sweden, which serves a population of 400,000–500,000.

### Study population

All inclusions were performed between January and April 2016. Individuals, born 1946 or earlier, that had been admitted to a ward of infectious diseases or internal medicine with ongoing intravenous treatment with cefotaxime, meropenem, or piperacillin-tazobactam since at least 24 h and able to provide informed written and oral consent, were approached for inclusion.

### Sampling and sample analysis

Two samples, at different time points, were drawn from each patient, primarily for internal validation. To try to achieve steady state-like trough concentrations, measurements on the first day of treatment were avoided. Samples were drawn shortly before the next scheduled antibiotic administration. Our aim was to have at least two doses of antibiotic between the first and the second sample, though in a few cases this was not achieved.

Following centrifugation at 1500×*g* for 15 min, the samples were stored at − 80 degrees Celsius awaiting analysis. Samples were analyzed at the Department of Clinical Pharmacology at Karolinska Institutet in Stockholm, using liquid chromatography-mass spectrometry (LC-MS) [15]. The results were given as total concentration of the antibiotic, which is the sum of the active, free concentration and the protein-bound fraction.

### Data collection and definitions

Information on baseline data, data from the ongoing infection as well as outcomes was collected from medical records.

Baseline data included information on the patients’ age, sex, weight, and height (body mass index, BMI), the reason for hospital admission, the Charlson/Deyo comorbidity index [16, 17] and current medication. Renal function was estimated using the Cockcroft-Gault equation for creatinine clearance (eGFR). Missing descriptive data were considered to be missing at random. Information about the ongoing infection included cultures taken at admittance and site of infection. In cases with defined etiology, the MIC for the causative agent was registered. Basic laboratory parameters such as C-reactive protein (CRP) and white blood cell count were also obtained. An assessment of sepsis severity [18] was made within 24 h of admission or at the start of infection (for nosocomial infections). Outcomes collected included days of hospital stay, 28-day mortality, and 28-day readmission, as well as cause of death and/or of readmission.

### Defining target intervals and categorization of antibiotic concentration results

Type of antibiotic, dose, and dosing interval, as well as time of initiation of treatment and number of doses prior to sampling was registered at inclusion. We also registered whether each patient was given the recommended dose according to Swedish guidelines, based on eGFR (Supplemental Table 1).

The protein binding of piperacillin and cefotaxime is approximately 30% and 35–40%, respectively [19, 20], while for meropenem, it is 2% [21]. Predictions of unbound concentrations from total concentration and prior knowledge of protein-binding generally correlate well for antibiotics with moderate protein-binding [22]. Considering the protein-binding of each antibiotic and that meropenem therapy in patients admitted to wards of internal medicine or infectious diseases in our region is predominantly used in immune-deficient individuals, we suggest target interval of total trough concentration starting at the level of the non-species-related breakpoint. In this, non-ICU cohort, we proposed an ideal target range for total trough concentrations at 1–5 mg/L for cefotaxime, 2–10 mg/L for meropenem, and 4–20 mg/L for piperacillin, respectively. All concentration values were categorized as low (beneath the ideal range), on target, high (5–10× the non-species-related breakpoint), or very high (> 10× non-species-related breakpoint).

### Data analysis

Data analysis was performed through analysis of descriptive data, estimations of target attainment as well as univariate and multivariate regression analyses of associations between trough concentration levels and clinical outcomes.

Baseline descriptive data were compared between patients receiving the three different antibiotics. The chi-square and Kruskal-Wallis tests were used for categorical and continuous variables, respectively. Categorical and mean trough concentration results for each patient were used in subsequent regressions. The target attainment for the full cohort as well as for each antibiotic was calculated, and compared between each antibiotic using chi^2^ tests. Outcome data were determined for the full cohort as well as for each antibiotic, and comparisons between groups were performed using the chi^2^ test for categorical outcome data and Kruskal-Wallis for continuous outcomes.

### Regression modeling

Univariate linear and polynomial regressions were performed to assess associations between antibiotic concentrations (continuous and categorical, respectively) and descriptive as well as infection-related predictors. Univariate regressions were performed to establish associations between patient outcomes and predictors.

For categorical data on antibiotic concentrations, multinomial multivariate regression models were fitted for the full cohort. A separate model was fitted for only individuals receiving the recommended antibiotic dose. For continuous outcome data on antibiotic concentrations, multivariate linear regression models were fitted separately for mean concentrations of the three antibiotics. Multivariate regression models were fitted for patient outcomes. All multivariate models were fitted using the purposeful selection algorithm, maintaining predictors with *p* < 0.1 or a coefficient-changing effect of > 20% in the final model [23].

### Ethical considerations

The study was approved by The Regional Ethical Review Board in Lund, Sweden (2015/709). All participation in the study was based on oral and written consent.

## Results

The study included 102 patients. From 88 of these patients, two samples were obtained, resulting in 190 samples. Of these patients, 72 individuals were treated with cefotaxime, 18 with piperacillin-tazobactam, and 12 with meropenem.

### Descriptive data

Baseline descriptive data for the full cohort sorted by antibiotic treatment is presented in Table 1. Fewer women than men (39%) were included in the study. The median age for the entire cohort was 80 years, while meropenem-treated patients were slightly younger (median 75 years). Differences, though not statistically significant, regarding eGFR were seen between groups. All patients with neutropenia (*n* = 5) were treated with meropenem. In the full cohort, the most common infection was pneumonia, affecting 33% of the patients (Supplemental Table 2).

**Table 1:** Baseline descriptive data on included patients

Variable	Full cohort 102 patients	Cefotaxime 72 patients	Meropenem 12 patients	Piperacillin 18 patients	Significant difference between any of the three groups
Gender % women (*n*)	39.2% (40)	44.4% (32)	41.7% (5)	16.7% (3)	*p* = 0.10
Age median (IQR)	80 (74–86)	82.5 (75–87.5)	75 (72.5–80)	80 (74–84)	*p* = 0.09
eGFR median (IQR) [MV]	50 (36–71) [3]	49.5 (37.3–68.8) [2]	60.5 (34.9–86.3) [0]	47 (31–71) [1]	*p* = 0.57
BMI median (IQR) [MV]	23.9 (21.5–27.3) [15]	23.5 (21.4–27.4) [13]	22.9 (20.9–27.3) [1]	24.5 (21.5–27.3) [1]	*p =* 0.98
Charlson/Deyo score Median (IQR)	3 (1–4)	3 (1–4)	3 (0.8–4)	3 (1.3–6)	*p* = 0.53
Each part of Charlson/Deyo comorbidity index % (*n*)					
Myocardial infarction	19.6% (20)	16.7% (12)	16.7% (2)	33.3% (6)	*p* = 0.27
Congestive heart failure	22.5% (23)	23.6% (17)	33.3% (4)	11.1% (2)	*p* = 0.33
Peripheral vascular disease	12.7% (13)	12.5% (9)	0	22.2% (4)	*p* = 0.20
Cerebrovascular disease	19.6% (20)	18.1% (13)	33.3% (4)	16.7% (3)	*p* = 0.44
Dementia	2% (2)	1.4% (1)	0	5.6% (1)	*p* = 0.46
Chronic lung disease	28.4% (29)	31.9% (23)	25% (3)	16.7% (3)	*p* = 0.42
Rheumatologic disease	19.6% (20)	20.8% (15)	16.7% (2)	16.7% (3)	*p* = 0.89
Peptic ulcer disease	2% (2)	1.4% (1)	0	5.6% (1)	*p* = 0.46
Mild liver disease	2% (2)	1.4% (1)	0	5.6% (1)	*p* = 0.46
Diabetes without organ damage	20.6% (21)	20.8% (15)	25% (3)	16.7% (3)	*p* = 0.85
Diabetes with organ damage	5.9%(6)	4.2% (3)	0	16.7% (3)	*p* = 0.09
Hemiplegia/paraplegia	5% (5)	5.6% (4)	0	5.6% (1)	*p =* 0.70
Moderate or severe kidney disease	12.7% (13)	13.9% (10)	25% (3)	0	*p* = 0.35
Tumor within the past 5 years	30.4% (31)	26.4% (19)	58.3% (7)	27.8% (5)	*p* = 0.39
Moderate/severe liver disease	1% (1)	0	0	5.6% (1)	*p* = 0.10
AIDS	0	0	0	0	*–*
Malignant tumor with metastasis	5.9% (6)	4.2% (3)	8.3% (1)	11.1% (2)	*p* = 0.50

*IQR* interquartile range, *MV* number of missing values

### Trough antibiotic concentrations

**Fig. 1:**
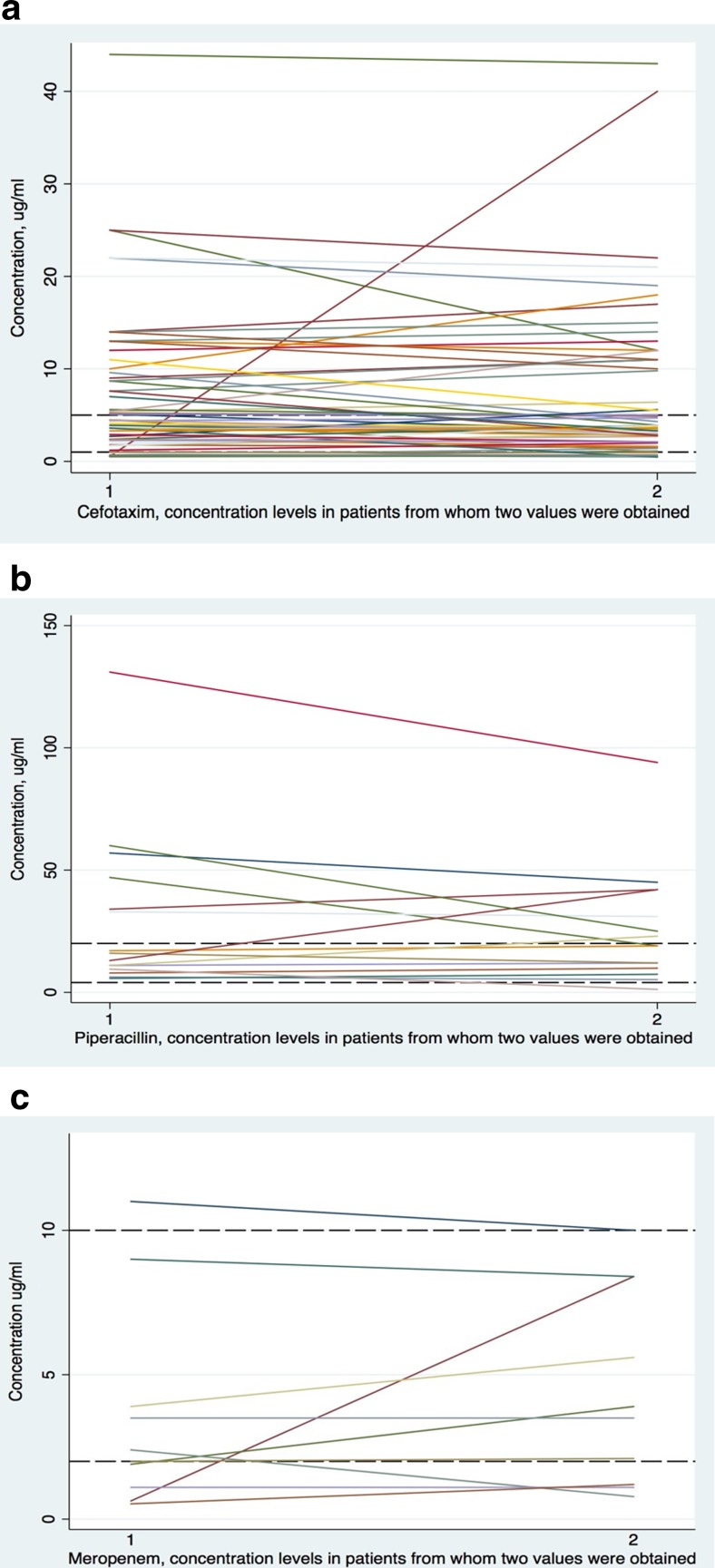
Spaghetti plots illustrating the variation in total trough antibiotic concentration levels in the patients from whom two values were obtained. The dashed lines mark the reference interval used for target concentration levels (non-species-related breakpoint − 5× non-species-related breakpoint). **a** Cefotaxime values (target interval used 1–5 mg/L or μg/mL). **b** Piperacillin values (target interval used 4–20 mg/L or μg/mL). **c** Meropenem values (target interval 2–10 mg/L or μg/mL)

Trough concentrations varied considerably in the group (Table 2). Only 36% of patients had concentration values within the defined target interval. Meropenem concentrations were generally low in relation to the target interval and three out of five patients with neutropenia had concentrations below the reference interval. Trough concentrations for piperacillin were often high. Only one patient receiving piperacillin-tazobactam had a low piperacillin concentration, and none had two low values. Trough cefotaxime concentrations were variable, as 42% of patients had at least one value above the target interval and 22% of patients had at least one value below the target interval. Limited variation between paired samples was observed (Fig. 1).

**Table 2:** Antibiotic concentrations in the study

Variable	Full cohort 102 patients	Cefotaxime 72 patient	Meropenem 12 patients	Piperacillin 18 patients	Significant difference between any of the three groups
Antibiotic concentration (mg/L) Mean (range) and median (IQR) [MV] 1. Concentration measurement 1 2. Concentration measurement 2	–	1. 6.1 (0–44) and 3.6 (1.3–7.9) [0]	1. 3.1 (0.53–11) and 1.95 (1–3.6) [0]	1. 34.5 (5.7–131) and 16.5 (9.9–43.8) [0]	–
		2. 7.0 (0–43) and 3.7 (2–11) [10]	2. 3.8 (0–10) and 2.8 (1–6.3) [0]	2. 25.8 (1.2–94) and 19 (11–36.5) [3]	
Proportion of patients not receiving recommended dose % (*n*)	19.6% (20)	23.6% (17)	16.7% (2)	5.6% (1)	*p* = 0.22
Proportion of patients with two trough values % (*n*)	86.3% (88)	84.7% (61)	100% (12)	83.3% (15)	–
Proportion of patients with all concentrations within the interval % (*n*)	36.3% (37)	36.1% (26)	33.3% (4)	38.9% (7)	*p* = 0.78
Proportion of patients with at least one low value % (*n*)	23.5% (24)	22.2% (16)	58.3% (7)	5.6% (1)	*p = 0.003*
Proportion of patients with two low values % (*n*)	9.8% (10)	8.3% (6)	33.3% (4)	0	*p = 0.008*
Proportion of patients with at least one high value % (*n*)	40.2% (41)	41.7% (30)	8.3% (1)	55.6% (10)	*p = 0.032*
Proportion of patients with two high values % (*n*)	23.5% (24)	26.4% (19)	0	27.8% (5)	*p* = 0.122
Proportion of patients with at least one very high value % (*n*)	24.5% (25)	25% (18)	0	38.9% (7)	*p* = 0.052
Proportion of patients with two very high values % (*n*)	12.7% (13)	15.3% (11)	0	11.1% (2)	*p* = 0.33

Significant *p* values are shown in italics

*IQR* interquartile range, *MV* number of missing values

### Univariate regressions of trough concentrations as outcomes of baseline predictors

In a linear univariate regression, higher trough cefotaxime concentrations were significantly associated with increasing age, decreasing eGFR, sepsis severity, and higher comorbidity index (Table 3). The univariate regression for piperacillin trough concentrations suggested an association between high concentrations and moderate/severe liver disease (based on a limited number of observations).

**Table 3:** Univariate and multivariate linear regressions for trough concentrations of cefotaxime, meropenem, and piperacillin as outcomes of baseline predictors

Variable	Univariate regression Cefotaxime	Multivariate regression Cefotaxime	Univariate regression Meropenem^1^	Univariate regression Piperacillin^1^
Age	β = 0.42 *p = < 0.001*	β = 0.31 *p = 0.002* (95% CI 0.12–0.51)	β = − 0.18 *p* = 0.29	β = − 0.27 *p* = 0.83
Gender	β = − 1.24 *p* = 0.48		β = − 0.26 *p* = 0.90	β = − 18 *p* = 0.43
eGFR	β = − 0.11 *p = 0.002*		β = − 0.05 *p* = 0.10	β = − 0.31 *p* = 0.21
BMI	β = − 0.14 *p* = 0.38		β = − 0.33 *p* = 0.24	β = − 0.69 *p* = 0.61
Charlson/Deyo comorbidity index	β = 0.95 *p = 0.032*		β = − 0.54 *p* = 0.22	β = 0.96 *p* = 0.73
Each part of Charlson/Deyo comorbidity index	Diabetes with organ damage β = 10.5 *p = 0.014* Moderate/severe kidney failure β = 8.15 *p = 0.001*	Diabetes with organ damage β = 9.94 *p = 0.005* (95% CI 3.13–16.8) Moderate/severe kidney disease β = 6.92 *p = 0.001* (95% CI 3.03–10.8)	Myocardial infarction β = 5.02 *p = 0.039*	Peptic ulcer disease β = 84.7 *p = 0.012* Moderate/severe liver disease β = 84.7 *p = 0.012*
Sepsis severity	β = 3.48 *p = 0.007*	β = 3.43 *p = 0.002* (95% CI 1.35–5.50)	β = − 1.06 *p* = 0.50	β = − 4.67 *p* = 0.68
Day of treatment	β = 0.27 *p* = 0.52		β = − 1.03 *p* = 0.14	β = − 1.79 *p* = 0.74
Dose interval	β = − 0.02 *p* = 0.94		β = 1.40 *p* = 0.07	β = 3.96 *p* = 0.38
Dose given according to guidelines	β = β = − 0–4.34 *p = 0.031*		β = − 4.27 *p* = 0.09	β = 5.30 *p* = 0.89

Significant *p* values are italicized

*β* beta-coefficient, *CI* confidence interval

^1^There are too few observations to motivate a multivariate regression

Using categorical concentration outcomes (Table 4) of the full cohort, significant associations were again seen between high concentration levels and increasing age, decreasing eGFR and sepsis severity. Increased sepsis severity was also associated with low concentrations. Thus, in patients with severe sepsis, antibiotic concentrations varied greatly, emphasizing the need for therapeutic drug monitoring in this group.

**Table 4:** Polynomial univariate and multivariate regressions for categorical outcomes of antibiotic concentrations in the full cohort and among the subset of individuals that received antibiotic doses according to eGFR-based guidelines

Variable	Full cohort—univariate analyses (compared to conc. in the correct interval) A Low conc. B High conc. C Very high conc.	Full cohort—multivariate model (compared to conc. in the correct interval) A Low conc. B High conc. C Very high conc.	Those who have received dose of antibiotics according to guidelines- multivariate model (compared to conc. in the correct interval) A Low conc. B High conc. C Very high conc.
Age	A. β = − 0.01 (*p =* 0.727) B. β = 0.08 (*p =* 0.063) C. β = 0.14 (*p = 0.002*)		
Gender	A. β = 0.55 (*p =* 0.320) B. β = − 0.59 (*p =* 0.329) C. β = − 0.01 (*p =* 0.986)		
eGFR	A. β = 0.01 (*p* = 0.348) B. β = − 0.23 (*p* = 0.072) C. β = − 0.07 (*p = 0.001*)	A. β = 0.01 (*p =* 0.449) (95% CI − 0.01–0.03) B. β = − 0.06 (*p = 0.004*) (95% CI − 0.1–(−)0.02) C. β = − 0.07 (*p = 0.002*) (95% CI − 0.12–(−) 0.03)	A. β = 0.01 (*p* = 0.558) (95% CI − 0.01–0.03) B. β = − 0.02 (*p* = 0.269) (95% CI − 0.04–0.01) C. β = − 0.06 (*p = 0.005*) (95% CI − 0.10–(−) 0.02)
BMI	A. β = 0.08 (*p* = 0.158) B. β = 0.06 (*p* = 0.265) C. β = − 0.05 (*p* = 0.297)	A. β = 0.08 (*p* = 0.254) (95% CI − 0.06–0.22) B. β = 0.15 (*p = 0.041*) (95% CI 0.01–0.29) C. β = 0.06 (*p* = 0.424) (95% CI − 0.09–0.21)	
Charlson/Deyo comorbidity index	A. β = − 0.24 (*p* = 0.093) B. β = − 0.01 (*p* = 0.952) C. β = − 0.05 (*p* = 0.694)		
Sepsis severity	A. β = 0.88 (*p = 0.039*) B. β = 0.76 (*p* = 0.072) C. β = 1.24 (*p = 0.005*)	A. β = 1.06 (*p = 0.034*) (95% CI 0.08–2.03) B. β = 0.68 (*p* = 0.203) (95% CI − 0.36–1.72) C. β = 0.88 (*p* = 0.109) (95% CI − 0.2–1.95)	A. β = 1.06 (*p = 0.025*) (95% CI 0.13–1.99) B. β = 0.73 (*p* = 0.155) (95% CI − 0.28–1.74) C. β = 0.49 (*p* = 0.380) (95% CI − 0.61–1.59)
Day of treatment	A. β = 0.25 (*p* = 0.208) B. β = − 0.31 (*p* = 0.278) C. β = 0.32 (*p* = 0.097)		
Dose interval	A. β = 1.17 (*p* = 0.289) B. β = − 0.94 (*p* = 0.141) C. β = − 1.02 (*p* = 0.113)		

Significant *p* values are italicized

*β* beta-coefficient, *CI* confidence interval

### Multivariate regressions of trough concentrations as outcomes of baseline predictors

Following adjustment for covariates, significant associations remained between increasing trough concentrations of cefotaxime and increasing age, diabetes with end organ damage, moderate/severe kidney disease, and higher sepsis severity (Table 3). Due to limited number of observations in the groups of meropenem and piperacillin, multivariate linear models were not fitted for these antibiotics.

The multivariate model for categorical outcomes of the full cohort (Table 4) revealed a significant association between a low eGFR and high concentrations. Low concentrations were significantly associated with increasing sepsis severity. In individuals given antibiotic doses according to guidelines, low eGFR remained significantly associated with very high concentrations, while increasing sepsis severity remained significantly associated with low concentrations (Table 4).

### Hospital stay, readmissions, and mortality

The median length of stay was 9 days for the entire cohort, while the 28-day mortality was 12.7% (*n* = 13). Patients treated with piperacillin-tazobactam had the highest mortality rate. The 28-day readmission rate was 22.5% (Table 5).

**Table 5:** Patient outcomes

Variable	Full cohort 102 patients	Cefotaxime 72 patients	Meropenem 12 patients	Piperacillin 18 patients	Significant difference between any of the three groups
Days of hospitalization Median (IQR)	9 (6–17)	8 (6–14)	10 (7.5–20.5)	14 (8–19)	*p* = 0.12
^2^Mortality within 28 days %	12.7% (13)	9.7% (7)	8.3% (1)	27.8% (5)	*p* = 0.09
^3^Readmission within 28 days %	22.5% (23)	23.6% (17)	16.7% (2)	22.2% (4)	*p* = 0.87
^3^Readmission within 28 days due to treatment failure	12.7% (13)	11.1% (8)	16.7% (2)	22.2% (4)	*p* = 0.45
Change of antibiotic within 48 h due to lack of effect	4.9% (5)	5.6% (4)	0% (0)	5.6% (1)	*p* = 0.70

*IQR* interquartile range

A significant association between high concentration of antibiotics and 28-day mortality as well as increased length-of-stay was observed (Table 6). As expected, a higher Charlson/Deyo score was significantly associated with 28-day mortality. No significant associations were seen between hospital readmission and included predictors.

**Table 6:** Multivariate outcome analyses for duration of hospitalization, hospital readmission, and mortality compared to patients within the target concentration interval

Variable	Duration of hospitalization	Readmission to hospital within 28 days	28-day mortality
Low concentration	β = 1.12 *p =* 0.81 (95% CI – 7.87–10.1)	OR = 0.60 *p =* 0.47 (95% CI 0.15–2.44)	–
High concentration	β = 12.01 *p = 0.01* (95% CI 3.00–21.0)	OR = 1.13 *p =* 0.84 (95% CI 0.33–3.87)	OR = 1.18 *p =* 0.85 (95% CI 0.19–7.25)
Very high concentration	β = 9.65 *p = 0.048* (95% CI 0.10–19.2)	OR = 0.91 *p =* 0.87 (95% CI 0.25–3.34)	OR = 5.68 *p = 0.027* (95% CI 1.22–26.46)
Charlson/Deyo comorbidity index	β^1^ = − 0.24 *p =* 0.75 (95% CI^2^ − 1.69–1.21)		OR = 1.47 *p = 0.005* (95% CI 1.12–1.94)
Age	β = − 0.43 *p =* 0.089 (95% CI^2^ − 0.92–0.066)		

Significant *p* values are italicized

*β* beta-coefficient, *CI* confidence interval, *OR* odds ratio

## Discussion

In this prospective observational study, total trough antibiotic concentrations of cefotaxime, meropenem, and piperacillin in older adults hospitalized with infection varied considerably. This variation was pronounced in individuals with severe sepsis. When a target interval of the non-species-related breakpoint − 5× the non-species-related breakpoint was applied, only 36% of patients had both values within the range. Most of the off-target concentrations of cefotaxime and piperacillin were above the target interval but significant inter-individual variation was evident. For meropenem, off-target concentrations were generally below the target interval. Even though the study was not powered to properly assess patient outcomes, dosing recommendations based on serum-creatinine-based renal function estimations clearly do not provide predictable trough concentrations of our most commonly used antibiotics in older adults, especially not in individuals with severe sepsis.

The major strength of this study is the relevance to standard of care. In the study, we have applied what we believe to be a feasible way of approximating *f*T > MIC. A large proportion of patients treated with intravenous antibiotics in hospitals are older adults with comorbidities, and in this group, infection-related mortality is increased [24]. However, the study also has limitations. The cohort size is limited and the study was not properly powered to assess consequences in patient outcomes. Being pragmatic in nature, the study was neither designed to measure exact *f*T > MIC nor to make proper pharmacokinetic simulations, which would require a larger number of samples per patient. Also, even though often used in clinical studies, to meet target intervals of trough concentrations of beta-lactam antibiotics has not been unambiguously demonstrated to correlate with improved clinical outcomes.

Due to the risk of inadequate treatment effect, low concentrations are the most immediate concern. For meropenem treatment, which in Sweden is reserved for the most severe infections and for patients with neutropenia, 100% *f*T > MIC is suggested. In the study region, the recommended dose of meropenem in infection was 500 mg×3, for patients with neutropenia 500 mg×4, and for patients with severe sepsis 1000 mg×3. More than half of individuals treated with meropenem had at least one low trough concentration value, suggesting that 500 mg (×3 or ×4) do not reliably provide 100% *f*T > non-species-related breakpoint. Increasing sepsis severity was associated with low as well as high trough concentrations, likely reflecting the timing of sepsis as well as the degree of renal involvement and hydration. Previous studies performed on patients with severe sepsis, mainly in ICU settings, have also demonstrated great variations in beta-lactam concentration levels [2, 25]. Roberts et al. have shown that 16% of critically ill patients did not achieve 50% *f*T > MIC and that these patients were less likely to have a positive clinical outcome [12], while Udy et al. demonstrated a 58% target attainment rate using a target concentration greater than or equal to MIC [26].

For many patients receiving cefotaxime and piperacillin-tazobactam, high trough concentration levels were seen. Dosage recommendations based on eGFR defined by Cockcroft-Gault alone may be too crude, and it is well described that alternative estimates of eGFR provide better guidance for older adults [27]. For a few patients receiving piperacillin-tazobactam, trough concentrations were very high (50–150 mg/L). This was significantly associated with liver disease. However, current recommendations do not suggest dose adjustment in patients with liver cirrhosis [20]. Overall, there is limited knowledge on the potential toxicity of high beta-lactam concentrations in humans. High concentrations may lead to reversible encephalopathy [28] and nephrotoxicity [29]. Threshold levels for beta-lactam concentrations where 50% of individuals develop adverse event have recently been suggested [30], but in older adults, significantly increased risk of adverse events is expected [7]. In the present study, an association between 28-day mortality and very high trough concentration levels was seen. However, the causality is unclear, and the association may be confounded by end-stage organ failure, despite adjusting for comorbidities.

In conclusion, current dosage guidelines for intravenous beta-lactam antibiotics do not provide predictable trough antibiotic concentrations in older adults hospitalized with infection. Better predictors are needed to guide antibiotic dosing in this group, and increased use of therapeutic drug monitoring of beta-lactams would be useful in patients with severe sepsis, where concentration levels were especially difficult to predict.

## Electronic supplementary material

ESM 1 (DOCX 51.7 kb)

ESM 2 (DOCX 16.3 kb)
