# Supplementary material for: Considerable variation of trough β-lactam concentrations in older adults hospitalized with infection—a prospective observational study
Source: Eur J Clin Microbiol Infect Dis. 2018 Jan 29;37(3):485–93. doi: 10.1007/s10096-018-3194-x (PMC5816762; doi:10.1007/s10096-018-3194-x)
Supplement: Supplementary file 1 — (DOCX 51.7 kb) [file 10096_2018_3194_MOESM1_ESM.docx]

**Supplemental Table 1.** Recommended dosing of included antibiotics during the study

| **Creatinine clearance (ml/min)** | **>80** | **41-80** | **20-40** | **<20** |
| --- | --- | --- | --- | --- |
| **Cefotaxime** | 1g x 3 | 1g x 3 | 1g x 2 | 1g x 2 |
| **Meropenem^1^** | 0.5g x 3 | 0.5g x 3 | 0.5g x 3 | 0.5g x 2 |
| **Meropenem high dose^2^** | 1g x 3 | 1g x 3 | 1g x 3 | 1g x 2 |
| **Piperacillin-tazobactam** | 4g x 3 | 4g x 3 | 4g x 3 | 4g x 2 |
| **Piperacillin-tazobactam**  **high dose^2^** | 4g x 4 | 4g x 4 | 4g x 4 | 4g x 3 |

^1^ In patients with neutropenia, 0.5g x 4 is recommended

^2^ Recommended for *Pseudomonas aeruginosa* infections.
