# Supplementary material for: Considerable variation of trough β-lactam concentrations in older adults hospitalized with infection—a prospective observational study
Source: Eur J Clin Microbiol Infect Dis. 2018 Jan 29;37(3):485–93. doi: 10.1007/s10096-018-3194-x (PMC5816762; doi:10.1007/s10096-018-3194-x)
Supplement: Supplementary file 2 — (DOCX 16.3 kb) [file 10096_2018_3194_MOESM2_ESM.docx]

**Supplemental Table 2** Descriptive data on infections

| **Variable** | **Full cohort**  **102 patients** | **Cefotaxime**  **72 patients 02** | **Meropenem**  **12 patients** | **Piperacillin**  **18 patients** | **Significant difference between any of the three groups** |
| --- | --- | --- | --- | --- | --- |
| **Sepsis severity Median* (IQR)^1^** | 1 (0-1) | 1 (0-1) | 1 (1-1.5) | 1 (1-2) | *p*=0.12 |
| **Pneumonia % (n)** | 33.3% (34) | 34.7% (25) | 25% (3) | 27.8% (5) | *p*=0.80 |
| **Urinary tract infection % (n)** | 19.6% (20) | 20.8% (15) | 8.3% (1) | 22.2% (4) | *p*=0.57 |
| **Wound infection % (n)** | 7.8% (8) | 5.6% (4) | 8.3% (1) | 16.7% (3) | *p*=0.29 |
| **Abdominal infection % (n)** | 3.9% (4) | 2.8% (2) | 8.3% (1) | 5.6% (1) | *p*=0.61 |
| **Other infection % (n)** | 8.8% (9) | 9.7% (7) | 16.7% (2) | 0% (0) | *p*=0.26 |
| **Unknown infection % (n)** | 26.5% (27) | 26.4% (19) | 33.3% (4) | 22.2% (4) | *p*=0.80 |
| **Neutropenic infection % (n)** | 4.9% (5) | 0% (0) | 41.7% (5) | 0% (0) | ***p*=<0.001** |

Significant p-values are bolded
^1^IQR= interquartile range
